# Correlation Baijiu analysis of physicochemical factors, microbial communities and flavor compounds during the piling process of Furou-type Baijiu

**DOI:** 10.3389/fmicb.2025.1683374

**Published:** 2025-10-20

**Authors:** Yanbo Liu, Huanhuan Geng, Yanyan Tang, Pengpeng Zhang, Mengjiao Xu, Hongliang Liu, Yonghua Wang, Huawei Li, Chunmei Pan

**Affiliations:** ^1^College of Food and Biological Engineering (Liquor College), Henan University of Animal Husbandry and Economy, Zhengzhou, China; ^2^Henan Province Engineering Technology Research Center of Liquor Style, Henan University of Animal Husbandry and Economy, Zhengzhou, China; ^3^Henan Province Brewing Special Grain Development and Application Engineering Research Center, Henan University of Animal Husbandry and Economy, Zhengzhou, China; ^4^Zhengzhou Key Laboratory of Liquor Brewing Microbial Technology, Henan University of Animal Husbandry and Economy, Zhengzhou, China; ^5^School of Life and Health Sciences, Hubei University of Technology, Wuhan, China; ^6^College of Biological Engineering, Henan University of Technology, Zhengzhou, China; ^7^Henan Caihongfang Distillery Co., Ltd., Xincai, China

**Keywords:** Furou-type Baijiu, physicochemical factors, microbial communities, flavor substances, correlation

## Abstract

In order to investigate the correlations among physicochemical factors, microbial communities, and flavor compounds during the fermentation process of Furou-type Baijiu, high-throughput sequencing (Illumina MiSeq platform) and Gas chromatography-mass spectrometry (GC-MS) were employed to analyze dynamic changes in microbial composition and flavor profiles. Redundancy analysis (RDA) and Spearman's correlation coefficients were applied to elucidate the interrelationships among these parameters. The results indicated that the relative abundance of *Weissella* gradually decreased, whereas that of *Acetobacter* increased over time. The dominant fungal genus shifted from *unclassified_o_Eurotiales* to Pichia. During the early fermentation stage (0–20 h), microbial community structure was strongly influenced by acidity and starch content. In contrast, at later stages (30 h and 40 h), temperature, moisture, reducing sugar, and pH exhibited greater impacts. The synthesis of alcohols and esters was significantly associated with variations in the microbial community. This study provides a theoretical foundation for optimizing the production process of Furou-type Baijiu.

## 1 Introduction

Chinese Baijiu is one of the world's six major distilled spirits. After several thousand years of development, it has played an irreplaceable role in various aspects such as society, economy, healthcare and diet (Shi et al., 2022; Xu et al., 2023). “Blending of aromas” is the trend of development for the production technology of Baijiu. Based on inheriting traditional techniques, continuous innovation is carried out. By drawing lessons from others and seeking breakthroughs, the quality of Baijiu can be enhanced, product styles can be diversified, and it can meet the diversified demands of the market and the changing tastes of consumers (Zhang et al., 2018; Su et al., 2017). Among them, Furou-type Baijiu is a natural and appropriate expression of the fusion of the four types of Baijiu aroma, thick, saucy, clear, sesame aroma, thick does not lose the sauce, the sauce does not pressure the thick, the aroma of the harmonic and natural, give people a more comfortable drinking experience, the taste is full, sweet, long aftertaste, the ripeness of the body, the sheer sweetness of the Baijiu, the style is unique, with a typical flavor characteristics of the Central Plains of the Baijiu flavor features and stylistic characteristics (Chen et al., 2022).

During the brewing process of Baijiu, pile fermentation is an important technique, which involves a complex microbial community and plays a crucial role in determining the flavor of the liquor as well as the yield and quality of the Baijiu. The accumulation of relevant microbial enzymes is necessary to ensure proper fermentation of the mash and the formation of flavor precursors (Wang et al., 2021). Bacterial properties are mainly on different kinds of enzymes such as proteases, amylases, esterases, etc., which produce abundant flavor substances and their precursors (Yang et al., 2015); Yeast mainly produces ethanol, esters and other various flavor substances; molds can produce enzymes such as saccharifying enzymes and liquefying enzymes, which are beneficial for the utilization of raw materials and the growth of special aroma-producing microorganisms (You et al., 2021). Furthermore, physical and chemical factors have significant influence on microbial communities (Guan et al., 2020). Therefore, studying the correlations among physicochemical factors, microbial communities and flavor substances during the maturation process of Furou-type Baijiu is of vital importance for improving the maturation process and enhancing the quality of Furou-type Baijiu.

In recent years, the piling process has been widely applied in the brewing of various types of Baijiu, such as strong-aroma, sauce-aroma, and light-aroma Baijiu. Previous researchers have conducted certain studies on this. Ren et al. (2023a,b) conducted a study on the correlation between the microbial community, physicochemical properties and flavor substances in the production of sauce-flavored Baijiu in Xiasha Lunding. The results indicated that starch was the main physicochemical property influencing the microbial succession during the pile fermentation process, *Lactobacillus, Weissella, Wickerhamomyces* and *Aspergillus* play key roles in the synthesis of metabolites; Zhou et al. (2024) conducted a study on the microbial communities and flavor substances during the first and second rounds of pile fermentation of the stone cave brewed sauce-flavored Baijiu (CBSB) by combining high-throughput sequencing technology with Gas chromatography-mass spectrometry (GC-MS) and liquid chromatography-mass spectrometry (LC-MS). The results indicate that, *Thermomyces, Torulaspora* and *Thermoascus* are the dominant fungal genus during the accumulation and fermentation process, Among them, *Virgibacillus, Kroppenstedtia* and *Bacillus* are the dominant bacterial genera. During the process of batch fermentation, 79 volatile metabolites were identified, mainly consisting of esters and alcohols. Li et al. (2022) selected Qingxiang Daqu and High temperature Daqu for piling up and tracked the fermentation process of Qingxiang-type Baijiu. They found that the total ester content of the fermented liquor after piling up was significantly higher than that of the unstacked liquor. Stacking could regulate the microbial community structure and promote the production of flavor substances. These studies have revealed the contributions of the piling up process to microbial succession and to flavor substances. Furou-type Baijiu has the flavor characteristics of integrating four types of Baijiu aromas: thick, saucy, clear, sesame aroma. As an innovative Baijiu, no previous studies have been conducted on it. Regarding the piling up process, most scholars have explored microbial community structure changes through high-throughput sequencing technology alone, or combined Illumina Miseq sequencing technology with Gas chromatography-mass spectrometry (GC-MS) to investigate microbial communities and volatile flavor substances. However, there are relatively few reports on simultaneously studying and analyzing the physicochemical factors, microbial communities, and volatile flavor substances during the piling up fermentation process of Baijiu. The understanding of the correlations among these three factors is not comprehensive enough. Therefore, this paper takes the Furou-type Baijiu produced by Henan Cai Hongfang Distillery as the research object. By using Illumina Miseq high-throughput sequencing technology and GC-MS technology, the microbial community structure succession rules and volatile flavor substances during the accumulation fermentation process of Fugrouxiang type Baijiu are analyzed. Based on redundancy analysis method (RDA) and Spearman correlation coefficient, the correlations among physicochemical factors, microbial community and flavor substances of Furou-type Baijiu are analyzed. This provides a more comprehensive exploration of the influence of accumulation fermentation on the fermentation process of Baijiu. It offers theoretical basis for studying the mechanism of accumulation fermentation and regulating microbial community, and provides scientific basis for improving the product quality, safety and production controllability of Furou-type Baijiu.

## 2 Materials and methods

### 2.1 Sample collection

The sample is sourced from Henan Cai Hongfang Liquor Co., Ltd, According to the fermentation time of accumulation, Select the sampling time points as 0 h, 10 h, 20 h, 30 h and 40 h. There are a total of five samples, and they are named as DJ0, DJ10, DJ20, DJ30 and DJ40. The fermented grains were piled up in a trapezoidal shape, samples were collected respectively at each sampling time point from the four sides of the piled-up fermented grains, collect samples from two points on each surface (10 cm away from the top layer) and 10 cm inward from the bottom layer. Mix them together to form a sample of the fermented grains for each surface. Then, mix the fermented grains taken from each surface evenly and take it as one sample to eliminate the sampling error. After mixing, immediately pack it in a sterile sealed bag and store it in a −80 °C refrigerator for long-term preservation after the sample collection is completed.

### 2.2 Method

#### 2.2.1 Determination of physicochemical indicators

Measurement of fermented grains temperature: While taking the sample, insert the thermometer into the exact center of the fermentation heap and keep it there for 1 to 5 min. After the reading of the thermometer stabilizes, record the data. According to DB34/T 2264-2014 “Analysis Methods for Solid-state fermented grains,” determine the moisture content, acidity, reducing sugar content and starch content.

pH measurement: It is directly measured by a precision pH meter. Sample pretreatment is required. The steps are as follows: Take 10 g of the mash sample (accurate to 0.01 g), place it in a 250 mL conical flask, accurately add 100 mL of water, stir well, and let it soak at room temperature for 30 min. During the soaking period, stir once every 15 min. Filter the extract using double-layer gauze or absorbent cotton, discard the initial filtrate of 20 mL, and take the filtrate for storage in the conical flask.

#### 2.2.2 DNA extraction followed by PCR amplification

The bacterial genomic DNA was extracted using the E.Z.N.A.^®^ Soil DNA Kit (Omega Bio-tek) in accordance with the operation procedures provided by the manufacturer. After extraction, the DNA concentration and purity (A260/A280 ratio) were determined using a NanoDrop 2000 spectrophotometer, and the integrity of the DNA was verified by 1% agarose gel electrophoresis.

The genomic DNA of fungi was extracted by the CTAB method. The extracted DNA was precipitated and dissolved in ultrapure water (100 μL) containing 10 ng/μL RNase, and incubated at 37 °C for 1 h to remove RNA contamination. Then, it was aliquoted and stored at −20 °C for future use.

PCR amplification: The V3-V4 variable region of the 16S rRNA gene was amplified using primers 338F 5′-ACTCCTACGGGAGGCAGCAG3′) and 806R 5′-GGACTACHVGGGTWTCTAAT3′). The amplification procedure was as follows: 3 min of pre-denaturation at 95 °C, 27 cycles (95 °C denaturation for 30 s, 55 °C annealing for 30 s, 72 °C extension for 30 s), then 10 min of stable extension at 72 °C, and finally preservation at 4 °C (PCR instrument: ABI GeneAmp^®^ 9700). The PCR reaction system was: 4 μL of 5 × TransStart FastPfu buffer, 2 μL of 2.5 mM dNTPs, 0.8 μL of upstream primer (5 μM), 0.8 μL of downstream primer (5 μM), 0.4 μL of TransStart FastPfu DNA polymerase, 10 ng of template DNA, and ddH_2_O to make up to 20 μL. Each sample had 3 replicates (Shanghai Meiji Biomedical Technology Co., Ltd.).

#### 2.2.3 Illumina Miseq sequencing

After mixing the PCR products of the same sample, the PCR products were recovered using 2% agarose gel. The recovered products were purified using AxyPrep DNA Gel Extraction Kit (Axygen Biosciences, Union City, CA, USA). The recovered products were detected and quantified using Quantus™ Fluorometer (Promega, USA) by 2% agarose gel electrophoresis. The library construction was carried out using NEXTflexTM Rapid DNA-Seq Kit (Bioo Scientific, USA): (1) linker ligation; (2) removal of self-ligated fragments by magnetic bead screening; (3) enrichment of library template by PCR amplification; (4) magnetic bead recovery of PCR products to obtain the final library. Sequencing was performed using Illumina's Miseq PE300 platform (Shanghai Meiji Biomedical Technology Co., Ltd.).

#### 2.2.4 Determination of volatile flavor compounds

Sample pretreatment: Take 2 g of the sample of the fermented grains, add 4 mL of 60% ethanol saturated brine solution, vortex for 30 s to mix evenly, ultrasonic for 5 min, centrifuge at 10,000 r/min for 5 min, take 2 mL of the supernatant, filter through a membrane, and add 3 μL of the mixed internal standard (tert-amyl alcohol, n-butyl acetate and 2-ethylhexanoic acid) before conducting gas chromatography analysis.

GC conditions: Injection port temperature 250 °C, carrier gas is high-purity helium (He) (>99.999%), total flow rate 50 mL/min, column flow rate 1.2 mL/min, purge gas flow rate for the septum 3 mL/min, injection mode is splitless injection. Temperature program: 40 °C for 2 min, increase by 3 °C/min to 180 °C, increase by 6 °C/min to 240 °C, hold for 5 min.

MS condition: Electron ionization (EI) source, electron energy 70 eV, ion source temperature 230 °C, quadrupole temperature 150 °C, mass scan range 27–330 amu.

### 2.3 Data processing

Each assay was performed three times and the results were described as mean and standard deviation, The data analysis and processing are carried out using Microsoft Office Excel 2016 software; the plotting of physicochemical index data is done with Origin 2024 software; the statistical analysis of volatile flavor data is conducted with SPSS19.0 software; the analysis of environmental microbial diversity and correlation heat maps is performed through the online platform of MajorBio Cloud (http://www.majorbio.com).

## 3 Results and analysis

### 3.1 Detection of physicochemical indicators during the fermentation process of Furou-type Baijiu

**Figure 1:**
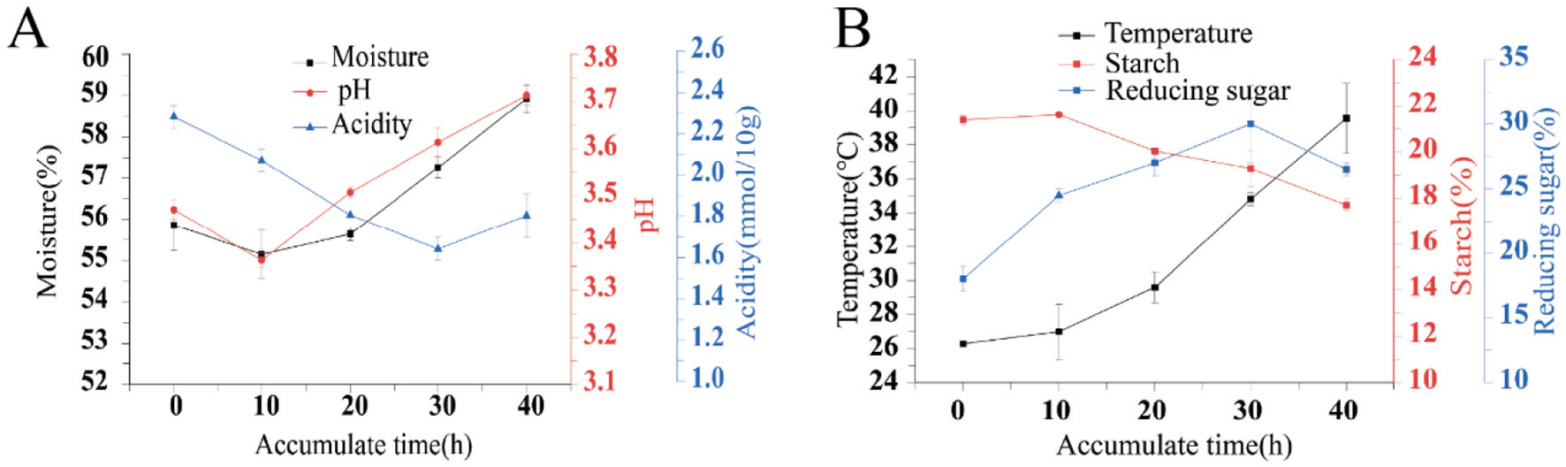
Test results of physicochemical indexes in the process of stacking grains. Moisture pH Acidity **(A)** and temperature Starch Reducing sugar **(B)**.

The physicochemical factors during the process of fermentation accumulation are important factors influencing the succession of microbial community structure. The moisture content of the fermented grains does not change significantly during the accumulation and fermentation process, remaining basically at 55% to 59%, and the overall trend is a decrease followed by an increase. The moisture content drops by 10 h, reaching the lowest value of 55.15%, and then it continues to rise slightly for the next 30 h; During the accumulation process, the acidity showed a slow downward trend, reaching the lowest value of 1.70 at 30 h. Then it began to rise, but the acidity of the fermented grains at the end of the accumulation (40 h of accumulation) was lower than that at the beginning of the accumulation (0 h of accumulation); pH, on the contrary, showed an overall upward trend, with pH decreasing from 0 to 10 h and reaching the lowest value of 3.37. From 10 h to 40 h, pH gradually increased and reached the peak value of 3.71 at 40 h (Figure 1A). During the fermentation process, the temperature of the fermented grains gradually increased throughout the process, starting at around 25 °C and ending at around 40 °C. Starch, as the substrate for microbial proliferation and metabolism, gradually decreased in content during the fermentation process. The content of reducing sugar gradually increased from 0 to 30 h, reaching a peak of 30%, and then began to decline after 30 h (Figure 1B).

### 3.2 Analysis of microbial diversity during the accumulation process of fermented grains

#### 3.2.1 Analysis of the diversity of alpha during the process of fermented grains accumulation

The diversity analysis of Alpha is usually employed to analyze the diversity of microbial communities in samples. The diversity analysis of a single sample, Alpha, can reflect the species abundance and diversity of microbial communities within the sample (Tian et al., 2020). As shown in Table 1, the sequencing coverage rates of both bacteria and fungi are above 0.99, indicating that the sequencing depth is sufficient and almost all samples in the samples have been detected. The sequencing results can truly reflect the diversity composition of bacterial and fungal communities in the fermented grains samples. As shown in Table 1, for bacteria, the Chao1 index decreased from 0 to 30 h, then gradually increased and decreased again from 30 to 40 h, and reached the highest value at 0 h of the piling up fermentation and the lowest value at 20 h of the piling up fermentation; for fungi, the Chao1 index kept decreasing. The Shannon index and Simpson index of bacteria remained basically unchanged; the Shannon index of fungi decreased slowly while the Simpson index remained basically unchanged. Overall, the richness and diversity of fungi were relatively high at the early stage of the accumulation of the fermented grains, and the richness of bacteria was generally higher than that of fungi.

**Table 1:** Analysis of microbial α diversity during piling up of fermented grains.

**Sample**	**Chao**		**Shannon**		**Simpson**		**Coverage**	
	**Bacteria**	**Fungus**	**Bacteria**	**Fungus**	**Bacteria**	**Fungus**	**Bacteria**	**Fungus**
DJ0_1	525.06	51.00	3.08	1.82	0.15	0.24	0.9980	0.9999
DJ0_2	568.59	47.00	3.26	1.50	0.13	0.34	0.9978	0.9999
DJ0_3	540.39	51.00	3.21	1.22	0.13	0.41	0.9979	0.9999
DJ10_1	501.67	45.60	2.87	1.55	0.20	0.33	0.9981	0.9999
DJ10_2	453.45	49.00	2.85	1.31	0.19	0.40	0.9982	0.9998
DJ10_3	488.44	44.50	3.21	1.34	0.14	0.38	0.9984	0.9999
DJ20_1	469.63	32.25	2.94	0.91	0.14	0.61	0.9979	1.0000
DJ20_2	437.48	32.50	2.99	1.52	0.16	0.29	0.9982	0.9999
DJ20_3	381.92	44.50	2.90	1.28	0.19	0.41	0.9988	0.9999
DJ30_1	504.11	42.00	3.16	1.21	0.14	0.42	0.9982	0.9999
DJ30_2	454.10	44.00	2.97	1.18	0.15	0.45	0.9982	0.9998
DJ30_3	486.51	29.25	3.13	1.13	0.14	0.42	0.9980	1.0000
DJ40_1	516.47	30.00	3.11	0.95	0.12	0.45	0.9980	0.9999
DJ40_2	464.47	26.33	2.60	0.93	0.19	0.46	0.9979	1.0000
DJ40_3	372.94	28.50	2.82	0.97	0.15	0.46	0.9987	0.9999

#### 3.2.2 The dynamic composition of microbial communities during the accumulation process

**Figure 2:**
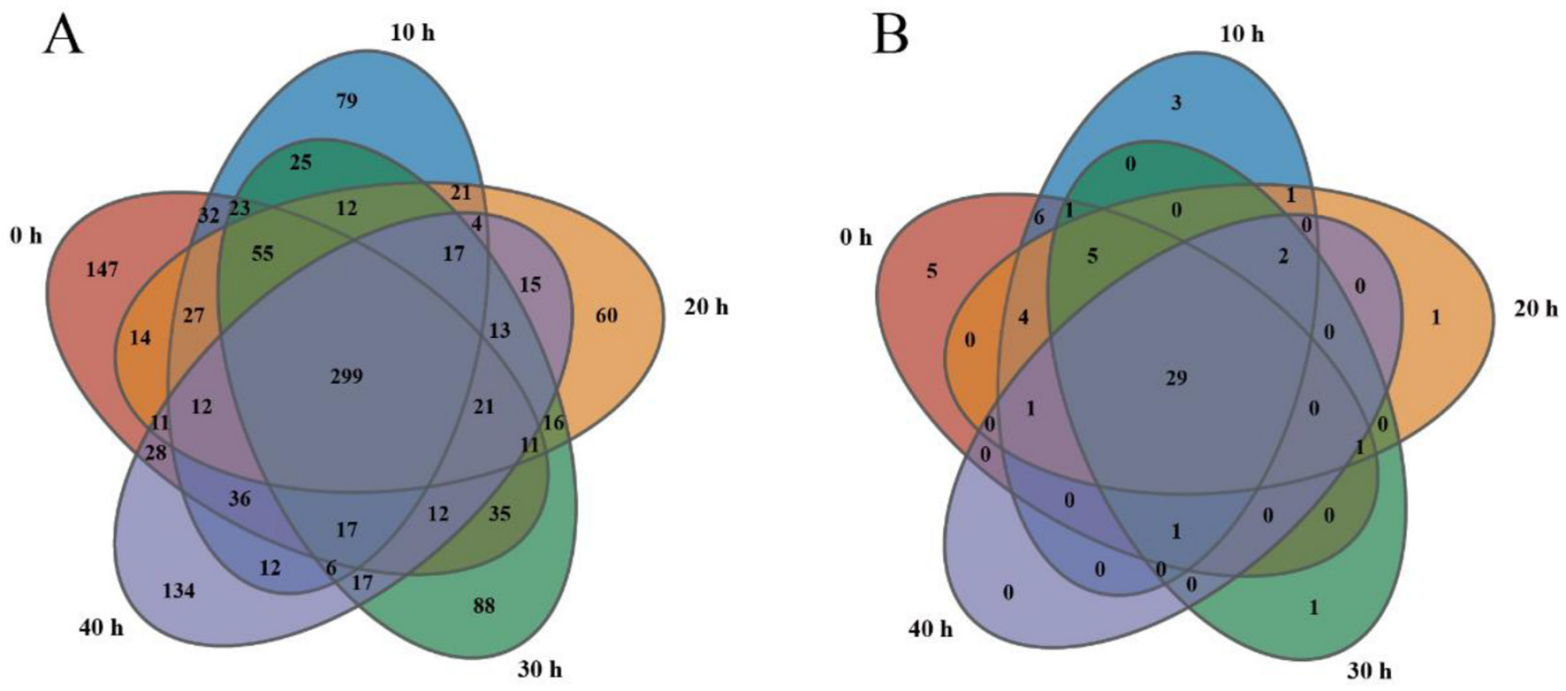
Venn diagram of bacteria **(A)** and fungi **(B)** OUT during the stacking of fermented grains.

As shown in Figure 2A, the bacterial OTUs of the samples accumulated at 0 h, 10 h, 20 h, 30 h, and 40 h were 780, 677, 608, 667, and 642, respectively. The total number of common OTUs was 299, and the OTUs specific to the samples in the five time periods were 147, 79, 60, 88, and 134, respectively. As shown in Figure 2B, the fungal OTUs of the samples accumulated at 0–40 h were 53, 53, 44, 40, and 34, respectively. The total number of common OTUs was 29, and the OTUs specific to the samples in the five time periods were 5, 3, 1, 1, and 0, respectively. The results show that the number of bacterial OTUs in the 0 h sample was the highest at 780, while the number of fungal OTUs in the 0 h and 10 h samples was the highest at 53. This is similar to the analysis results in Table 1. During the accumulation process, the number of OTUs of bacteria and fungi differed the least at 10 h and 30 h, and the greatest difference occurred at 0 h and 20 h.

#### 3.2.3 Diversity of microbial community structure

**Figure 3:**
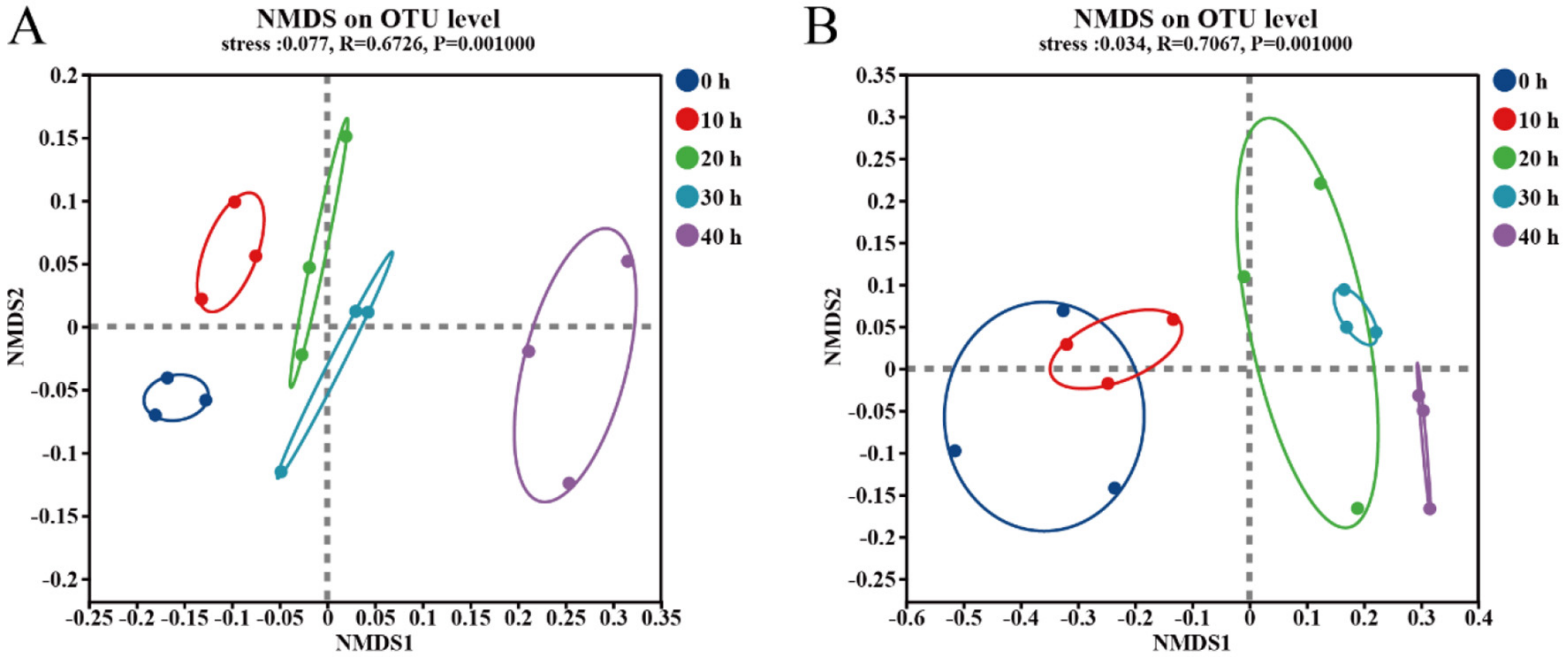
NMDS analysis of bacteria **(A)** and fungi **(B)** in five accumulation stages based on Bray-Curtis distance.

In order to study the diversity of microbial structure during the fermentation stage of the fermented grains, NMDS analysis was conducted on samples collected from different accumulation times. As shown in Figure 3, with the passage of accumulation time, the community structure of bacteria and fungi is constantly evolving. There are overlapping parts among the samples of fungi at different time points, indicating that there are shared microbial communities during the accumulation process. For bacterial communities, there is a trend of community succession in the accumulated samples. The samples accumulated at the 40th hour showed a significant separation from those accumulated in other time periods, indicating that the bacterial communities are significantly different. For the samples collected at the 0 h and 10 h of the fungal community accumulation, as well as those collected at the 20 h and 30 h, there were overlapping parts. This indicates that there are shared microbial communities among them.

### 3.3 Analysis of microbial community structure during the accumulation process of fermented grains

Based on the results of Illumina Miseq sequencing, the data were classified at the phylum and genus levels. The genera and phyla with an average relative abundance of > 1.00% were defined as the dominant genera and phyla.

**Figure 4:**
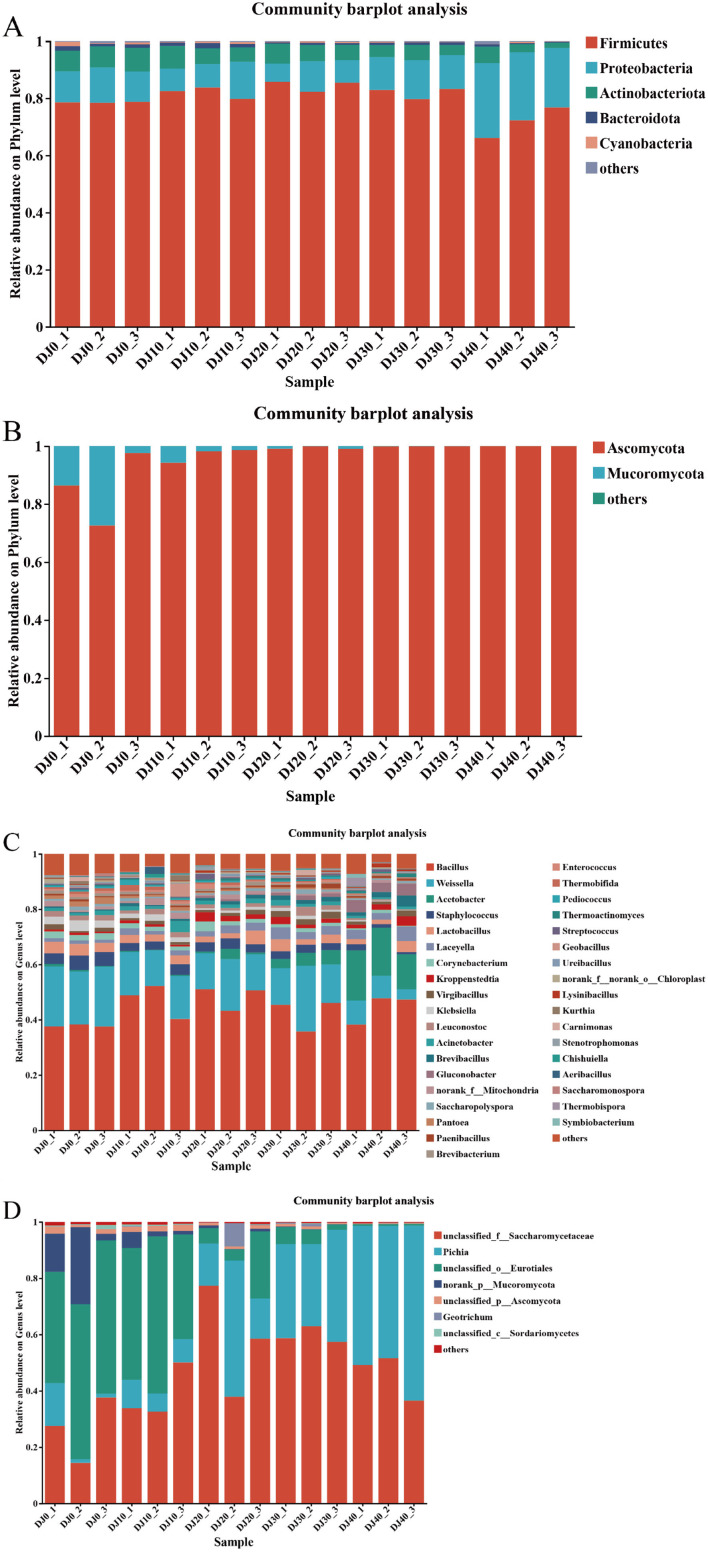
Microbial community structure during the stacking of fermented grains. Microbial community structure at phyla level bacteria **(A)** and fungi **(B)**. Microbial community structure at genus level bacteria **(C)** and fungi **(D)**.

At the phylum level, four bacterial phyla and one fungal phylum were identified (Figure 4A). The dominant bacterial phyla include Firmicutes, Proleobacteria and Actinobacteriota, etc. During the fermentation process, Firmicutes are dominant, with an average relative abundance of 83.27%, The third group is Proleobacteria, Actinobacteriota and Bacteroidota, with their average relative abundances being 11.83%, 3.57% and 1.05% respectively. During the piling up fermentation process, the Proleobacteria phylum gradually increases while the Actinobacteriota phylum gradually decreases. At the fungal phylum level (Figure 4B), the dominant fungal phylum is Ascomycota. During the piling up fermentation process, Ascomycota always holds a dominant position, with an average relative abundance of 99.85%.

During the piling up and fermentation process of the Furou-type Baijiu, the bacterial genera detected in the ermented grains were 14 in number, and the dominant fungal genera were 4 in number (Figure 4). The dominant bacterial genera include *Bacillus* (46.12%), *Weissella* (13.90%), *Acetobacter* (5.20%), *Laceyella* (3.20%), *Lactobacillus* (3.12%), *Staphylococcus* (2.45%), etc. (Figure 4C). Among them, the average relative abundance of *Bacillus* remained relatively stable throughout the accumulation process and always maintained a dominant position. At 0 h of the fermentation process, the average relative abundance of *Weissella* was 21.64%. After 40 h of the fermentation, the average relative abundance dropped sharply to 8.16%, while *Acetobacter* showed an upward trend, with its average relative abundance increasing from the initial 0.25% to 17.40%. During the fermentation process, *Staphylococcus* showed a slow decline trend, dropping to 1.26% at 40 h of the fermentation time. The detected fungal genera include *Pichia, unclassified-f-saccharomycetaceae, Geotrichum*, and *unclassifided-o-Eurotiales* (Figure 4D). The dominant fungal genus is *Pichia*. During the 0–20 h accumulation process, the average relative abundance of *unclassified-f-saccharomycetaceae* remained between 37 and 50%. After 30 h of accumulation, the average relative abundance suddenly increased to 62.9%, and then dropped sharply to 51.61% after 40 h of accumulation. At the beginning of the batch fermentation, the dominant microorganisms were *unclassified-f-saccharomycetaceae* and *unclassifided-o-Eurotiales*. After 30 h of fermentation, the relative abundance of *unclassifided-o-Eurotiales* decreased, and the dominant microorganisms changed to *unclassified-f-saccharomycetaceae* and *Pichia*. When the fermentation was carried out for 20 h, the average relative abundance of *Pichia* rose from 8.29% at 10 h to 48.41% at 20 h, then dropped to 29.16% at the 30th h, and rose to 47.02% at the end of the fermentation. Meanwhile, the average relative abundance of *unclassifided-o-Eurotiales* decreased sharply from 39.55% at the beginning of the fermentation (0 h) to 3.99% at the end of the fermentation, and remained below 10% throughout the fermentation process.

### 3.4 Analysis of the correlation between advantageous microorganisms and physical-chemical factors

**Figure 5:**
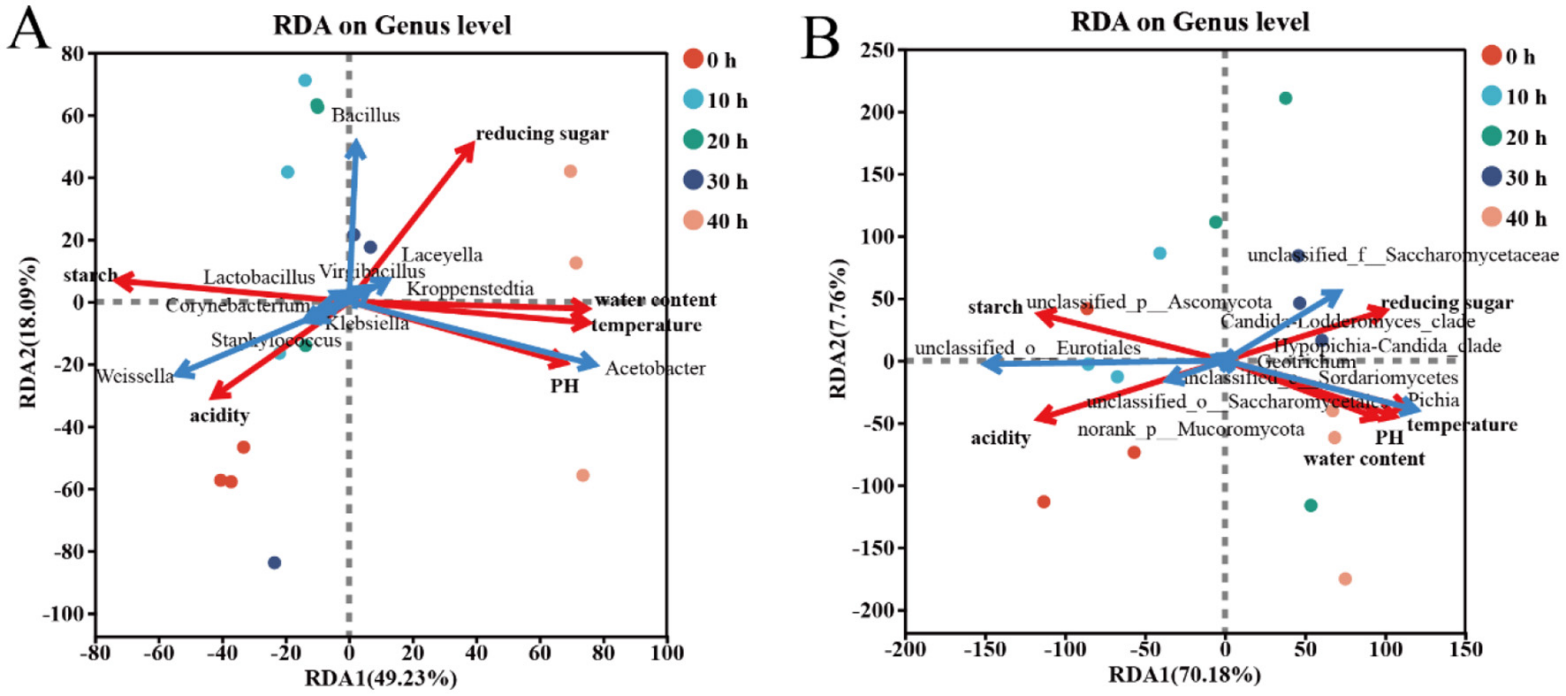
RDA analysis between microbial communities and physicochemical factors of the top 10 dominant microorganisms at the genus level. Bacterial Community **(A)** Correlation with fungal communities **(B)** and physicochemical factor. Different colored dots represent different sample groups. Positive correlation: The angle between the physicochemical factors is acute; negative correlation: The angle between the physicochemical factors is obtuse.

Through redundancy analysis (RDA), the mutual influence between physicochemical factors and dominant bacterial genera in the accumulated fermented grains of Furou-type Baijiu was revealed (Figure 5). The two axes respectively accounted for 67.32 and 77.94% of the differences in community composition. Starch and acidity were negatively correlated with temperature, moisture, pH and reducing sugar. According to the redundancy analysis (RDA), it was found that the most influential factor for the bacterial community structure during the stationary fermentation stage was acidity at 0 h. From 10 to 20 h, the influence of starch and acidity was significant, from 30 h, the influence of reducing sugar was significant, and from 40 h, the influence of temperature, moisture, pH and reducing sugar was significant. Among the beneficial bacteria genera, *Acetobacter* showed extremely significant positive correlations with temperature, moisture and pH (*p* < 0.001), and significant positive correlations with reducing sugar (*p* < 0.05), while showing extremely significant negative correlations with acidity and starch (*p* < 0.01). In contrast, *Staphylococcus* was exactly the opposite of *Acetobacter*, and *Weissella* showed significant negative correlations with temperature (*p* < 0.05). The structure of the fungal community was greatly influenced by starch and acidity during the period of 0–10 h. During the period of 20–30 h, it was greatly affected by temperature, moisture, reducing sugar and pH. During the period of 40 h, it was greatly influenced by temperature, pH and moisture. The genus *Pichia* of the family Saccharomyces cerevisiae is positively correlated with temperature, water content, pH and reducing sugar, and negatively correlated with acidity and starch; while *unclassifided-o-Eurotiales* is positively correlated with acidity and starch, and negatively correlated with temperature, water content and pH.

### 3.5 The volatile flavor compounds during the fermentation process of Furou-type Baijiu

A total of 26 volatile flavor compounds were detected. Among them, there were 6 alcohols, 6 esters, 7 acids and 7 others (Table 2). Phenylethyl Alcohol, 2,3-Butanediol, 2-Furanmethanol and 3-Methyl-1-butanol are the higher alcohols in the distillery mash samples. In this study, the content of Phenylethyl Alcohol increased from 0.72 mg/kg at 0 h to 10.30 mg/kg at 40 h; the content of 2,3-Butanediol kept increasing and reached 1.26 mg/kg at 40 h of fermentation; 2-Furanmethanol was not detected until 40 hours of accumulation; And 3-Methyl-1-butanol also emerged at the mid-stage of the accumulation process, and its content gradually increased during the accumulation process. During the entire accumulation process, the contents of Acetic acid, Butanoic acid and Hexanoic acid all increased initially and then decreased. The acetic acid content reached its maximum at 20 h, being 9.17 mg/kg, and remained relatively high throughout the entire process. Ethyl lactate, Ethyl caprylate and Methyl oleate can all be detected throughout the process of the mash pile-up, while Ethyl Acetate is detected only in the later stage of the pile-up; The contents of methyl oleate and ethyl lactate gradually increased with the piling up time, reaching the maximum at 20 h of piling up and then decreasing; The content of Ethyl caprylate keeps increasing. Methyl icosanoate was detected at 30 h of accumulation, with a content as high as 13.45 mg/kg.

**Table 2:** Results of determination of volatile flavor substances during the piling up of fermented grains.

**Compound**	**Content (mg/kg)**				
	**0 h**	**10 h**	**20 h**	**30 h**	**40 h**
2-Furanmethanol	–	–	–	–	1.37 ± 0.12a
Phenylethyl Alcohol	0.72 ± 0.22c	1.45 ± 0.84c	2.19 ± 0.16c	6.60 ± 2.30b	10.30 ± 0.29a
3-Methyl-1-butanol	–	–	1.45 ± 0.17c	5.07 ± 1.74b	7.42 ± 0.62a
Palmitoleyl alcohol	6.89 ± 5.75b	40.72 ± 20.08a	7.20 ± 3.74b	6.62 ± 4.37b	8.16 ± 10b
2,3-Butanediol	0.18 ± 0.02b	0.46 ± 0.27b	0.52 ± 0.06b	1.10 ± 0.62a	1.26 ± 0.07a
(Z)-hexadec-11-en-1-ol	14.07 ± 7.58a	–	–	–	–
Ethyl Acetate	–	–	1.14 ± 0.14b	3.97 ± 1.70a	3.73 ± 0.50a
Ethyl lactate	1.82 ± 0.28b	4.11 ± 2.28b	4.99 ± 0.63a	4.49 ± 0.37a	4.25 ± 0.36b
Ethyl caprylate	1.04± 1.24ab	0.60 ± 0.37b	1.21 ± 0.13ab	2.14 ± 0.66a	2.25 ± 0.53a
Methyl oleate	14.70 ± 1.39a	3.72 ± 3.14c	8.66 ± 0.24b	6.46 ± 1.60bc	6.86 ± 1.12b
Methyl icosanoate	–	–	–	13.45 ± 7.00a	–
Isopropyl palmitate	–	–	0.63 ± 0.07a	–	–
Acetic acid	2.84 ± 0.36c	9.17 ± 5.12a	8.03 ± 0.98ab	4.66 ± 1.62abc	4.27 ± 0.20bc
Butanoic acid	0.89 ± 0.15d	2.95 ± 0.62a	2.53 ± 0.40ab	1.52 ± 0.10c	1.93 ± 0.21bc
Hexanoic acid	1.24 ± 0.41a	2.92 ± 1.69a	2.89 ± 0.75a	2.31 ± 0.82a	2.28 ± 0.31a
Octadecanoic acid	16.48 ± 23.45a	2.86 ± 1.92a	22.10 ± 26.75a	40.37 ± 58.36a	13.45 ± 11.15a
n-Hexadecanoic acid	9.64 ± 2.62a	17.08 ± 10.00a	11.40 ± 4.95a	22.22 ± 9.35a	14.32 ± 5.10a
2-Methyl-butanoic acid	22.16 ± 0.15a	18.00 ± 3.72b	16.88 ± 0.25bc	14.72 ± 0.21c	16.43 ± 0.84bc
2-ethyl-Heptanoic acid	–	–	0.77 ± 0.33b	–	1.96 ± 0.25a
4-tert-Butylphenol	0.68 ± 0.13c	1.29 ± 0.75b	–	–	2.15 ± 0.12a
2,4-Di-tert-butylphenol	6.31 ± 2.07b	11.35 ± 6.41ab	10.80 ± 1.85ab	13.73 ± 3.07a	14.28 ± 1.74a
Retinal	0.00	11.13 ± 1.12ab	9.82 ± 8.78ab	14.28 ± 12.21a	2.30 ± 0.37ab
Benzenepropanal	0.55 ± 0.17b	1.00 ± 0.57ab	0.82 ± 0.07ab	1.29 ± 0.43a	1.30 ± 0.19a
Phenyl cyclohexyl ketone	0.56 ± 0.16b	1.18 ± 0.22a	0.79 ± 0.12ab	1.11 ± 0.38a	1.18 ± 0.22a
Acetoin	–	–	–	4.39 ± 1.81b	5.95 ± 0.23a
1,2,4-trimethyl-Benzene	0.74 ± 0.44bc	0.60 ± 0.37bc	2.55 ± 1.16a	–	1.46 ± 0.18b

Different letters in the same row indicate significant differences (*p* < 0.05), and “–” this indicates that the specific flavor substance was not detected at this accumulation time point.

### 3.6 The correlation between microorganisms and volatile compounds

**Figure 6:**
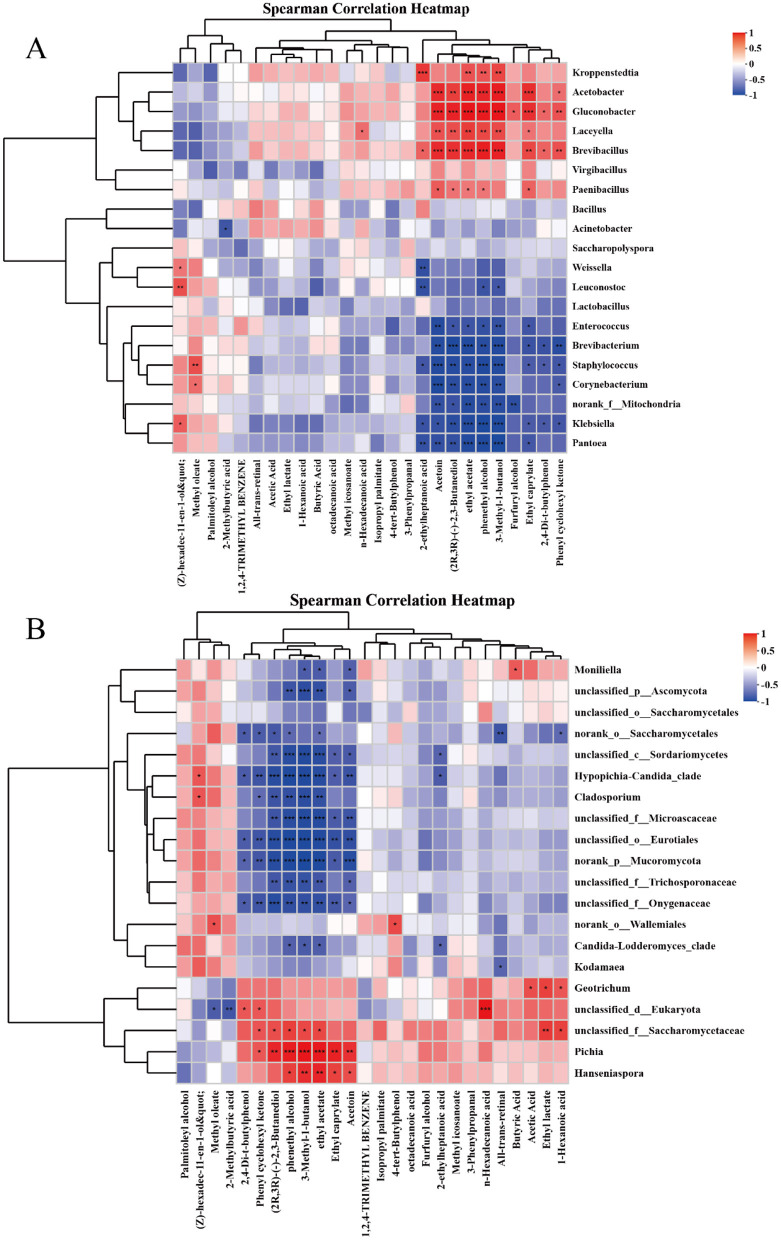
Heat map of the relationship between flavor compounds and dominant strains. Heat map of correlation between bacteria and flavor compounds **(A)** Heat map of correlation between fungi and flavor compounds **(B)**. If the p-value is less than 0.05, it will be marked with an asterisk (*) to indicate that the difference in correlation is significant; *p < 0.05, **p < 0.01, ***p < 0.001.

In order to explore the correlation between the microbial community and volatile flavor compounds in Furou-type Baijiu. The dominant microorganisms at the genus level (the top 20) were selected, and the Spearman correlation coefficient was used to analyze their correlations with flavor compounds (Figure 6). For the genus of bacteria, *Acetobacter* was significantly positively correlated with Ethyl caprylate, Phenylethyl Alcohol, 2,3-Butanediol, and Ethyl Acetate (*p* < 0.01), *Staphylococcus* showed a significant positive correlation with Methyl oleate (*p* < 0.05), and an extremely significant negative correlation with all 9 flavor substances (*p* < 0.01); *Brevibacillus* has a highly significant correlation (*p* < 0.01) with Ethyl caprylate, 3-Methyl-1-butanol, Phenylethyl Alcohol, Ethyl Acetate, and 2,3-Butanediol. *Weissella s*howed a highly significant positive correlation with 2-ethyl-heptanoic acid (*p* < 0.01), and a significant negative correlation with (Z)-hexadec-11-en-1-ol (*p* < 0.05). For the genus of fungi, *Pichia* has an extremely significant positive correlation with Phenylethyl Alcohol, Ethyl Acetate, and other alcohols and esters (*p* < 0.01); *Unclassified-f-saccharomycetaceae* was significantly positively correlated with Hexanoic acid, Ethyl Acetate, 3-Methyl-1-butanol, Phenylethyl Alcohol, 2,3-Butanediol (*p* < 0.05), it shows a highly significant positive correlation with ethyl lactate (*p* < 0.01); *Geotrichum* was significantly positively correlated with Acetic acid, Ethyl lactate, and Hexanoic acid (*p* < 0.05); The *unclassifided-o-Eurotiales* species showed a nearly negative correlation with all the substances.

## 4 Discussion

This study explored the correlations among physicochemical factors, microbial communities, and flavor substances during the fermentation process of Furou-type Baijiu. During the accumulation process, the temperature and moisture of the fermented grains overall show an upward trend. The temperature rise is likely attributable to microbial metabolic activity and associated biological heat generation (Xiao et al., 2017), a phenomenon consistent with observations in sauce-flavor Baijiu fermentation (Hao et al., 2021). In contrast, acidity dynamics diverged from previously reported trends (Ren et al., 2023a,b), potentially due to accumulation of organic acids derived from anaerobic microbial proliferation (Cai et al., 2019). Fermentation involves the coordinated microbial utilization and decomposition of substrates such as starch, yielding brewing enzymes and diverse metabolic products (Shan et al., 2016). Starch content decreased consistently, while reducing sugar increased initially within the first 30 h before declining by the 40th hour, likely due to early amylase-mediated starch hydrolysis followed by microbial consumption of sugars for ethanol and other metabolite synthesis (Dong et al., 2022).

By analyzing the microbial community structure of the fermented grains during the fermentation process, at the phylum level, a total of 4 dominant bacterial phyla and 1 dominant fungal phylum were detected (Figure 4). The beneficial bacterial phyla include Firmicutes, Proleobacteria, and Ascomycota, among others. The results of previous studies on the dominant microorganisms in the fermented grains of other types of Baijiu are basically consistent with this (Ren et al., 2023a,b; Wang et al., 2021; Song et al., 2017). Through redundancy analysis (RDA), the mutual influence between physicochemical factors and dominant bacterial genera in the fermented grains of Furou-type Baijiu was revealed (Figure 5). At the microbial level, Firmicutes dominate during the batch fermentation process. They have a strong ability to adapt to the environment and can maintain growth and metabolism under relatively extreme conditions (Wolf et al., 2004), At the genus level, the dominant genera are *Bacillus, Weissella, Acetobacter*, and *Pichia* (Figures 4C, D), which are consistent with the research results of the saccharifying and fermenting process of sauce-flavored Baijiu (Wu et al., 2013). However, the *Weissella* genus is the dominant genus during the saccharification and fermentation process of Furou-type Baijiu. It shows a significant negative correlation with temperature (*p* < 0.05). During the accumulation of fermented grains, the temperature keeps rising, while the average relative abundance of the *Weissella* genus decreases, Moreover, the *Weissella* genus has been discovered to be capable of producing antibacterial compounds and extracellular polysaccharides, which is beneficial for the food industry (Teixeira et al., 2021). *Bacillus* bacteria can secrete various enzymes to break down the fermentation substrate, thereby enabling their growth and the production of Baijiu-flavor substances (Cai et al., 2019; Xiao et al., 2021). The genus *Acetobacter* has a highly significant negative correlation with acidity and starch (*p* < 0.01). In contrast, the genus S*taphylococcus* has the opposite relationship. During the fermented grains, the acidity shows a downward trend. The average relative abundance of the genus *Acetobacter* is higher in the later stage of the accumulation process, while the average relative abundance of the genus *Staphylococcus* continuously decreases during the accumulation process. This indicates that high acidity inhibits the growth of other bacteria, but is conducive to the growth of the genus *Acetobacter*. *Virgibacillus* can secrete hydrolases, such as amylase, to hydrolyze the large molecular components in grains, especially during starch saccharification (Wang et al., 2014). *Pichia* exhibited significant positive correlations with temperature, moisture, pH, and reducing sugar, while showing a negative correlation with acidity and starch. In contrast, *unclassified_o_Eurotiales* was positively correlated with acidity and starch and negatively correlated with temperature, moisture, and pH. These opposing correlation patterns reflect the distinct ecological niches occupied by the two taxa and further explain the successional shift in the dominant fungal genus from *unclassified_o_Eurotiales* to *Pichia* during the later stages of pile fermentation.

Flavor compounds are produced by microorganisms, or are catalyzed by enzymes secreted by microorganisms during the brewing process (Dong et al., 2022). A total of 26 volatile flavor compounds were detected in the samples. Among them, there were 6 alcohols, 6 esters, 7 acids, and 7 other types (Table 2). Alcohol-based substances are crucial for creating a refreshing wine body. Appropriate amounts of higher alcohols can enhance the flavor and taste of Baijiu (Han et al., 2014). 2,3-Butanediol is a key substance that affects the caramel flavor and burnt aroma of the Baijiu (Wang, 2021); 2-Furanmethanol has a fusel oil-like aroma; 3-Methyl-1-butanol has a caramel-like sweet aroma (Wang et al., 2022); Phenylethyl Alcohol is a compound with a gentle, pleasant and long-lasting rose-like fragrance (Li et al., 2024). During the fermentation process, Saccharomyces cerevisiae can be catalyzed by a series of enzymes to synthesize β-phenylethanol (Zhu et al., 2021), This might be the reason for the increase in Phenylethyl Alcohol content in the later stages of accumulation. Fatty acids are of vital importance to the flavor of Baijiu, microorganisms can synthesize low-molecular-weight fatty acids such as Butanoic acid, Valeric acid, Hexanoic acid and Octanoic acid, which are precursors of ester compounds (Wei et al., 2020). The average relative abundance of *Acetobacter* is higher in the later stage of fermentation, its metabolic products mainly consist of Acetic acid, which can combine with substances such as ethanol produced during the cellar fermentation process, enriching Ethyl Acetate in the wine body (Han et al., 2021), it shows a highly significant positive correlation with Ethyl caprylate, phenylethanol, 2,3-butanediol, and ethyl acetate (*p* < 0.01), this might be the reason for the increase of these flavor substances in the later stage of accumulation. During the brewing process of Baijiu, ester substances are mainly formed through esterification reactions between acids and alcohols, as well as through the metabolism of microorganisms. They make a special contribution to the flavor of Baijiu (Jiang, 2020). The increase in the content of later-stage esters may be related to the accumulation of ester precursors and the metabolic activities of microorganisms (Xu et al., 2022). The variation in the content of Ethyl lactate may be related to the decrease in the average relative abundance of lactic acid bacteria in the microorganisms over time. Pichia, a non-Saccharomyces yeast, serves as a functional microorganism capable of producing significant quantities of alcohols and esters (Zhang et al., 2021). It has a highly significant positive correlation with various alcohols and esters such as Phenylethyl Alcohol and Ethyl Acetate (*p* < 0.01). 2,4-Di-tert-butylphenol shows an upward trend throughout the accumulation process, although it is only a small part of the accumulation process, it is an antioxidant and beneficial to human health (Rao et al., 2018).

These findings underscore the critical role of microbial succession and physicochemical interactions in shaping the distinctive flavor profile of Furou-type Baijiu. The dominance of *Weissella* and *Acetobacter*, along with the dynamic shifts between *unclassified_o_Eurotiales* and *Pichia*, highlight fermentation stage-specific microbial contributions to aroma and taste. Controlling temperature and acidity appears essential to guiding microbial activity and optimizing flavor compound formation. This study provides a scientific basis for improving fermentation management and consistency in Furou-type Baijiu production.

## 5 Conclusion

In this study, high-throughput sequencing technology was employed, combined with GC-MS technology, to analyze the microbial community, physicochemical properties and volatile flavor substances during the maturation process of Furou-type Baijiu, and to investigate their correlations. In terms of community succession, in the later stage of accumulation, *Acetobacter* replaced *Weissella* and became one of the dominant microorganisms; *Pichia* replaced *unclassifided-o-Eurotiales* and became the dominant microorganism. A total of 26 volatile compounds were detected during the fermentation process of the fermented grains. The main compounds that played a dominant role were alcohols and esters. The redundancy analysis showed that the microbial community structure during the 0–20 h of the fermentation process was significantly influenced by acidity and starch, while at 30 h and 40 h, it was greatly affected by temperature, moisture, reducing sugar, and pH. *Acetobacter* and *Pichia* showed extremely significant positive correlations with temperature, moisture, and pH (*p* < 0.001), and also showed significant positive correlations with reducing sugars; *Staphylococcus* showed extremely significant positive correlations with starch and acidity (*p* < 0.01). The correlation between the microbial community and volatile flavor substances was analyzed using the Spearman correlation coefficient. The results showed that *Acetobacter* was significantly positively correlated with caproic acid Ethyl caprylate, Phenylethyl Alcohol, 2,3-Butanediol, and Ethyl acetate (*p* < 0.01). This research is conducive to a scientific understanding of the Furou-type Baijiu. It provides a theoretical basis for further exploration of the correlations among physical and chemical factors, microbial communities, and flavor substances during the accumulation and fermentation process of the Furou-type Baijiu, and offers new parameters for improving the quality of the Furou-type Baijiu.

## Data Availability

The original contributions presented in the study are publicly available. This data can be found here: http://www.ncbi.nlm.nih.gov/bioproject/1322064.
